# Web review: Breast imaging

**Published:** 2009-05

**Authors:** IK Indrajit

**Affiliations:** Department of Radiodiagnosis and Imaging, Command Hospital (Air Force), Bangalore-560 007, India

A few useful websites related to breast imaging are reviewed below:

1. **Mammography Physics and Technology for Effective Clinical Imaging** provides educational material focusing on the technical aspects of mammography and its optimization. Available at http://www.sprawls.org/resources/MAMMO/module.htm, the material is authored by Perry Sprawls Ph.D. It deals with a host of technical issues, such as image quality characteristics, image exposure histogram, mammography technology, receptor and display systems, imaging technique factors, contrast sensitivity, film contrast factors, x-ray penetration and contrast, controlling the x-ray spectrum, the molybdenum spectrum, and molybdenum/rhodium filters.
2. **Mammography Tutorials** is an interactive site dealing with the analysis of mammograms. This web project from McGill Faculty of Medicine, which is coordinated by Dr. David Fleiszer, Montreal General Hospital, is available at http://sprojects.mmi.mcgill.ca/Mammography/index.htm. Broadly, the tutorials are on how to position and view a mammogram and interpret it. Included are topics such as anatomy, positioning, viewing the mammogram, mammogram analysis, asymmetric density, masses, calcifications, and an interactive mammography analysis web tutorial.
3. **Basic Principles of Mammographic Tissue Characterization** is a part of the board review notes from Creighton University and is accessible at http://radiology.creighton.edu/ and http://radiology.creighton.edu/boards/mmg2.htm. There are sections that cover the basic physics of mammography, breast composition determination, image quality, triangulating for lesion location, risk factors for breast cancer, noninvasive breast cancer, benign neoplasia of breast, benign breast processes, differential considerations for breast disease, and needle localization
4. **Notes, Scales, and Music** is a ‘web-based electronic tool that teaches mammographic skills.’ This novel site is featured at http://www.imagingdomain.com/charlie/notesscalesmusic-breast/Index.htm and is created by A. Davidoff, P. Slanetz, C. Allison, L. Wiechmann, and J. Makris. The website is structured on a model akin to the teaching of music, with three important sections: Notes, Scales, and Music. Specifically, ‘Notes’ initially explores the anatomical aspects, ‘Scales’ teaches and allows the learner to practice examination techniques, while ‘Music’ tests the ability of the learner to detect and, later, to classify and diagnose mammographic abnormalities.
5. **The Breast Module** at http://www.imagingdomain.com/rsna05/ is a web resource that has educational images. Created by A. Davidoff, it has wide ranging contents that covers the history, histology, anatomy, pathology, radiology, and clinical aspects of breast lesions. It has an image library consisting of over 1000 images. The offered topics range from applied biology, diseases, imaging modalities, findings, strategies, educational tools, PowerPoint library, and online links.
6. **Benign Breast Disease and Breast Cancer Tutorial** is a tutorial designed for the medical student. Available at https://mywebspace.wisc.edu/wwolberg/breast/, this site is authored by William H. Wolberg, M.D., from the University of Wisconsin. It deals with the normal breast anatomy and physiology, the presenting symptoms of breast cancer, the differential diagnosis of a breast mass, the identification of high-risk groups, genetic considerations, the management of a mammographically suspicious but nonpalpable lesion, breast cancer prognosis, the role and type of definitive surgery in breast cancer, and adjunctive therapy.
7. **BI-RADS^®^** representing ‘Breast Imaging Reporting and Data System’, is an important topic in Breast Imaging. The American College of Radiology offers detailed online information on the BI-RADS system at http://www.acr.org/SecondaryMainMenuCategories/quality_safety/BIRADSAtlas.aspx. A few important atlas excerpts from RADS^®^ *MRI*, BI-RADS^®^ *Ultrasound*, and BI-RADS^®^ *Mammography* are available at http://www.acr.org/secondaryMainMenuCategories/quality_safety/BIRADSAtlas/BIRADSAtlasexcerptedtext.aspx.
8. **BI-RADS Introduction to the Breast Imaging Reporting and Data System** is a brief outline created by Harmien Zonderland from Leiden University Medical Centre, Netherlands. The concisely compiled material is available at http://www.radiologyassistant.nl/en/4349108442109 and it covers the BI-RADS report organization, breast imaging lexicon, mammographic breast composition, and the final assessment of lesions.
9. **BI-RADS^®^ Breast Imaging Reporting and Data System Tutorial** is available at http://www.birads.at/index.html. This web-based tutorial is authored by Christopher C. Riedl, MD, G. Pfarl, MD, and T.H. Helbich, MD, from the Department of Radiology at the University of Vienna. It teaches ‘the mammographic characteristics of breast lesions according to this system’ by providing more than 400 histologically proven cases.
10. **Digital Mammography Research** from Purdue University is a condensed educational site authored by Professor Edward J. Delp. The site available at http://www.ece.purdue.edu/~ace/mammo/mammo.html deals with computer-aided diagnostic techniques for the automatic detection and classification of breast tumors. The topics featured include ‘Breast Abnormalities in Mammograms,’ ‘Statistical Segmentation of Mammograms,’ ‘Multiresolution Detection of Spiculated Lesions,’ and ‘Identification of Normal Digital Mammograms.’

## Endpiece

**Breast Cancer** at http://www.rsna.org/Education/archive/breast.cfm is a subsection of the RSNA Education Portal. The webpage acts as a junctional area, linking to important articles covering breast imaging that have been featured in the journal Radiographics. It also provides links to breast lesions featured in a ‘case of the day’ format.

An interactive computer-teaching module for **Radiological Pathological Correlations in Breast Imaging** written by Katherine E Maturen, MD, *et al*. from the University of Michigan is available at http://www.med.umich.edu/rad/b-imaging/edu/MammoWEB.swf.

A handy and useful link to **Mammography Databases** is available from Purdue University at http://www.ece.purdue.edu/~ace/mammo/mammo_db.html. It includes a Digital Mammography Database sourced from Mammographic Image Analysis Society, Washington University, Digital Database for Screening Mammography (DDSM), Nijmegen University, and UCSF/LLNL Library.

The **American Roentgen Ray** Society at http://www.arrs.org/ ‘is the first and oldest radiology society in the United States.’ The organization is ‘dedicated to the goal of the advancement of medicine through the science of radiology and its allied sciences.’ Its principal education tools range from the premier publication American Journal of Roentgenology to the quarterly magazine ARRS In Practice, both of which are linked from the ARRS Web portal.

